# Embracing complexity in plant–microbiome systems

**DOI:** 10.1111/1758-2229.70000

**Published:** 2024-08-27

**Authors:** María Josefina Poupin, Bernardo González

**Affiliations:** ^1^ Laboratorio de Bioingeniería, Facultad de Ingeniería y Ciencias Universidad Adolfo Ibáñez Santiago Chile; ^2^ Center of Applied Ecology and Sustainability (CAPES) Santiago Chile; ^3^ Millennium Nucleus for the Development of Super Adaptable Plants (MN‐SAP) Santiago Chile

## Abstract

Despite recent advances in understanding the role of microorganisms in plant holobiont metabolism, physiology, and fitness, several relevant questions are yet to be answered, with implications for ecology, evolution, and sustainable agriculture. This article explores some of these questions and discusses emerging research areas in plant microbiomes. Firstly, it emphasizes the need to move beyond taxonomic characterization towards understanding microbial functions within plant ecosystems. Secondly, controlling methodological biases and enhancing OMICS technologies' standardization is imperative for a deeper comprehension of plant–microbiota interactions. Furthermore, while plant microbiota research has primarily centred on bacteria and fungi, other microbial players such as archaea, viruses, and microeukaryotes have been largely overlooked. Emerging evidence highlights their presence and potential roles, underscoring the need for thorough assessments. Future research should aim to elucidate the ecological microbial interactions, their impact on plant performance, and how the plant context shapes microbial community dynamics. Finally, a discussion is provided on how the multiple layers of abiotic and biotic factors influencing the spatiotemporal dynamics of plant–microbiome systems require in‐depth attention. Examples illustrate how synthetic communities and computational methods such as machine learning and artificial intelligence provide alternatives to tackle these challenges and analyse the plant holobiont as a complex system.

## Graphical abstract

Microbiomes are vital for plant health, productivity, and ecology, yet many aspects still need clarification. Key issues include: How can we shift from focusing on taxonomy to studying functions? How can we reduce methodological biases? How do we account for overlooked microorganisms? How do microbe–microbe interactions affect plant performance? How can we analyse these interactions as complex systems? This article explores these questions and offers insights to advance our understanding of plant–microbe interactions.

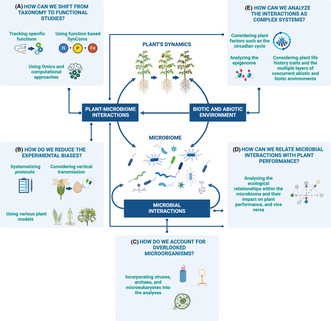

## INTRODUCTION

The recent realization of how intertwined the macroorganisms are with their microbiomes has challenged biology and evolution disciplines. Growing evidence is establishing connections between microorganisms and core elements of host biology, ecology, and evolution (e.g., Davenport et al., 2017; Hacquard et al., 2015; Haney et al., 2015; Pieterse et al., 2016; Sherwin et al., 2019). Further, it has been proposed that animals and plants should be considered autonomous entities composed of the host and its associated microbes (i.e., holobionts, Bordenstein & Theis, 2015; Margulis, 1993). Hiltner described the importance of plant‐associated microorganisms in plant health and productivity more than 100 years ago. He observed a ‘rhizosphere effect’, finding that the number of microorganisms associated with the plant roots was far higher than in the surrounding soil (Hartmann et al., 2008). We now know that microorganisms inhabit the plant rhizosphere and colonize their leaves, shoots, internal tissues, and seeds (Hallmann et al., 2006; Wallace, 2023). Additionally, the plant microbiota impacts the survival and growth of the hosts, two critical fitness components (Vandenkoornhuyse et al., 2015). Even though the rhizosphere effect was already known in the 60s, it was not considered during the ‘First Green Revolution’ (Pingali, 2012). However, the current need for sustainable agriculture, coupled with global change challenges, has increased interest in comprehending the benefits of using microorganisms for agricultural management (Batista & Singh, 2023; Pieterse et al., 2016).

Investigating the mechanisms governing interactions between organisms from different domains is usually complex, hindering the practical application of microbiota attributes from the laboratory to the field (Russ et al., 2023; Sessitsch et al., 2023). Over the past few years, researchers have focused on three main questions to study plant–microbiome interactions: Who are the microbiota members? What do they do? How do they do what they do? Thus, most of the studies of plant–microbiome systems have followed two principal strategies, mainly to assess the first two questions: (i) Correlational: Analysing the microbial composition of different plant microbiomes with metagenomic analyses to correlate it with changes in plant behaviour and health under different environmental scenarios and (ii) functional but reductionist: To analyse the role of specific members of a microbial community in the growth, development, or health of particular plant species. Now, it is well established that to enable the development of new and reliable microbiota‐based solutions to the different challenges that plants face, plant microbiome research should move into an era of causality with coordinated and cross‐disciplinary efforts. Here, we will discuss different issues and still open questions that should be considered to foster the movement in that direction. Additionally, we propose some venues to cover such questions.

*How can we better connect microbial taxonomy to function in plant microbiota research?*

Most plant–microbiome systems studies are based on the determination of the taxonomic composition of the microbiota, i.e., the bacterial, fungal, and rarely archaea and microeukaryotes composition (Bai et al., 2022; Chen et al., 2022a; Fitzpatrick et al., 2020; Jana et al., 2022; Poupin et al., 2023). The taxonomical characterization of plant microbiota has been mainly performed by collapsing strains with identical 16S rRNA in Operational Taxonomic Units. Nevertheless, strains from the same units can have functional differences due to variations in gene content (Shalev et al., 2022). This can be produced, for instance, by horizontal gene transfer, which reflects that 16S rRNA sequences evolve slower than the rest of the genome (Shalev et al., 2022). Amplified sequence variants have recently allowed deeper taxa definitions (Lucaciu et al., 2019; Lundberg et al., 2021). However, new techniques must enable finer taxonomical differentiation and absolute abundance determinations. Furthermore, the main pitfall of these reports is that they only provide a rather rough idea of what microbial functions may play a role in the plant holobiont (Compant et al., 2021). Of course, whole genome sequencing, or methodologies based on average nucleotide identity or similar, provides better data on genomic functions or taxonomical information but requires substantially more work than the 16S rRNA‐based approaches, diminishing studies´ coverture.

Despite the methodological difficulties, taxonomic data offer a general idea of the critical functions present in plant‐associated microbial communities. As a few examples, the presence of Actinomycetota suggests plant growth promotion, biocontrol activity, phosphate turnover, and antimicrobial synthesis; the (alpha class) Pseudomonadota: N fixation legume symbiosis, plant DNA integration, plant growth promotion; the (gamma class) Pseudomonadota: etabolic versatility, plant pathogen, biocontrol, plant growth promotion, pollutant degradation, bioactive molecules synthesis; the fungal phylum Ascomycota: versatile bioactive compounds synthesis, plant growth promotion, plant pathogen (Poupin et al., 2023, and therein supporting information references).

However, several reports indicate that taxonomical markers, in general terms, poorly predict function. For example, we have reported that at the biome scale, traits such as redox, among others, are better indicators of the performance of microbial communities than taxonomical markers, as revealed by meta‐transcriptomic analyses (Ramírez‐Flandes et al., 2019). At the species levels, genome comparisons provide a clearer picture of gene functions involved in microbial adaptation to plants (Levy et al., 2018). Therefore, additional studies reporting gene expression, protein patterns, and metabolic changes in the microbiota under different scenarios are required to clarify plant–microbiome interactions at the functional level. Some examples of relatively limited scope are already available, such as a microbial metaproteomic of the Arabidopsis phyllosphere (Delmotte et al., 2009) or a metaexoproteomic of the rhizosphere of *Brassica napus* L. (oilseed rape) (Lidbury et al., 2022). However, the potential of such approaches is still not fully explored (Armengaud, 2023).

Synthetic Communities (SynComs) are purposefully designed microbial consortia that mimic natural microbial interactions and functions while simplifying the complexity of natural microbiomes (Marín et al., 2021). SynComs can also help to understand the potential connection between functionality and taxonomy (Delgado‐Baquerizo, 2022; Vorholt et al., 2017). Several reports utilizing SynComs have evidenced that some effects on, for instance, microbial community structure are explained by specific strains within a species (see, for example, Finkel et al., 2020). For instance, studies have shown that commensal *Pseudomonas* strains can induce plant protection from pathogenic *Pseudomonas*, eliciting specific plant transcriptional responses not triggered by the pathogens themselves (Shalev et al., 2022). In other words, a key species role within a microbial network can be played by one strain but not by closely related ones, highlighting the importance of strain‐level considerations. In this context, it should be remembered that most SynComs are designed based on taxa abundance and representation on a sample or by identifying co‐occurrence patterns between taxa (Jing et al., 2024; Vorholt et al., 2017). This approach could introduce a taxonomical bias in these otherwise powerful experimental and conceptual tools. For example, Beilsmith et al. (2021) found that the colonization of roots, shoots, and leaves‐associated microbial communities varied in space and time, depending on bacterial traits not commonly shared within the same family. The design of SynComs based on functional representation (i.e., Fe mobilization, P uptake, N fixation, phytohormone synthesis, and degradation, volatile organic compounds production, antagonist (antimicrobial) activity, induction of systemic disease resistance, among others) would improve our knowledge on plant‐associated microbial community functions (i.e., Feng et al., 2023; Wang et al., 2021). The integration of computational methods, including machine learning and artificial intelligence, into SynCom design, promises intentional and efficient processes, thus streamlining the selection of SynComs for plant testing (Emmenegger et al., 2023; Gonçalves et al., 2023; Zhang et al., 2022). For instance, Emmenegger et al. (2023) used machine learning algorithms to assist the strain selection in SymComs designed to reduce pathogen colonization in *A. thaliana*. They found that the identity of the strains significantly impacted pathogen reduction. Additionally, the strains identified by machine learning were more effective than randomly selected strains.

**FIGURE 1:**
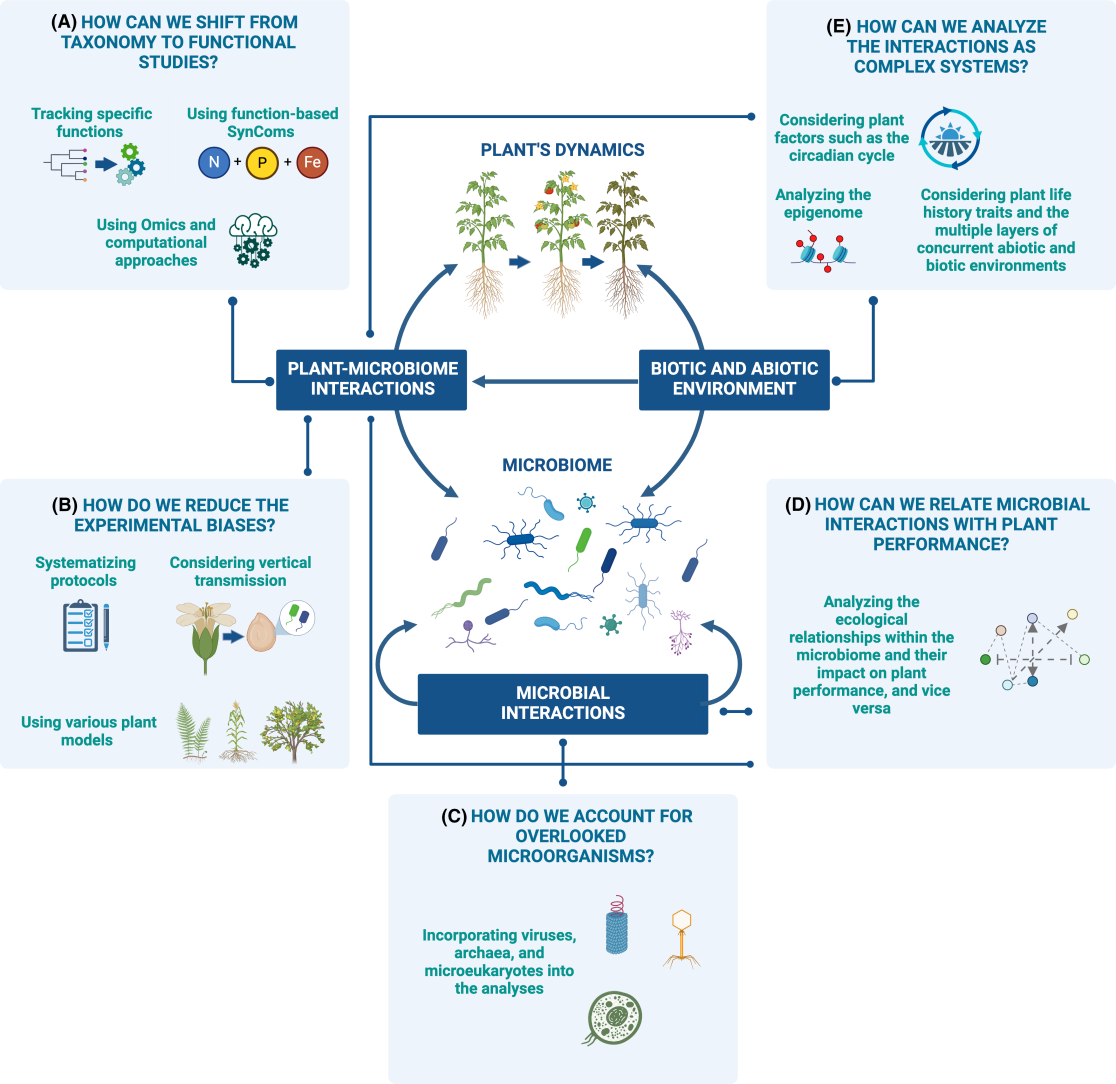
Multiple interactions in the plant holobiont. The holobiont comprises the host plant and its microbiome (centre). Many interactions occur within and between the components of the holobiont and the environment (centre). These interactions collectively impact the performance of the individual components and the holobiont (dotted connectors). (A) Since taxonomic characterization does not fully represent microbiome functions, incorporating OMIC techniques like genomics, transcriptomics, and proteomics is essential. These methods help uncover and understand microbial functions that impact host performance and track specific microbial functions. Additionally, designing SynComs based on member functionalities, such as biofertilizer strains, would be beneficial. (B) Plant holobiont research faces biases like PCR primers targeting specific microorganisms and variations in bioinformatic pipelines. Standardizing processes is crucial for comparability, considering the vertical transmission of strains in seeds and using diverse plant models. (C) Part of the knowledge of the microbiome roles in plants is only correctly addressed if we consider a broader range of microorganisms. (D) We must understand how competition and other ecological interactions impact natural communities and their role in plant fitness traits. (E) Plant and their microbiomes are complex systems composed of interacting parts where the outputs are not the sum of the parts, and multifactorial approaches should be addressed. This includes considering factors such as the circadian cycle, epigenetics, and the multiple layers of biotic and abiotic factors. Figure created with BioRender.com.

Genome‐scale metabolic networks (GSMNs) are also starting to be used in SynCom design, becoming a valuable tool for detecting biological functions relevant to the performance of a microbial community in plants. These networks encompass all metabolic reactions and pathways in microbial genomes, allowing for a comprehensive understanding of how these microorganisms contribute to overall ecosystem functions (Gonçalves et al., 2023; Mataigne et al., 2022). Additionally, GSMN reconstruction can help identify the minimal community capable of producing essential metabolites for productive plant–microbial interactions. Although algorithms and computational methods are promising for optimizing SynCom design, their use is still limited by the scarcity of large datasets. Furthermore, it is necessary to experimentally confirm the associations' validity (Jing et al., 2024). Therefore, bridging the gap between microbial taxonomy and function in plant microbiota research requires a multi‐faceted approach beyond traditional taxonomic characterization. By embracing advanced techniques, focusing on functional insights, leveraging SynComs, and integrating computational methods, we can better understand the complex interactions within plant‐associated microbial communities (Figure 1A and centre).

In synthesis, the connection between microbial taxonomy and function can be substantially improved by tracking specific, key microbial functional features, an appropriate combination of omics approaches, and continuing improvements of SynComs' design, helped by artificial intelligence technologies (Figure 1A).

### Are there methodological biases in the study of plant microbiota?

Microbiota studies are currently based on the use of next‐generation sequencing approaches. Variable experimental choices are observed in sampling procedures (including specific plant micro‐zones), targeted 16S rRNA sequence sites, PCR primer sequences and conditions, and DNA sequence amplification technologies (Figure 1B). For example, in Arabidopsis, we have found that thirteen different primer pair combinations targeting 16S rRNA have been used (Poupin et al., 2023). A couple of PCR primer sequences were compared in a few cases, with one showing less bias than the other (e.g., Lundberg et al., 2012; Thiergart et al., 2020). In addition, different DNA sequence data analyses, data bioinformatics pipelines, statistical packages, and alignment and taxonomical databases are utilized. All these differences, previously noted (e.g., Schlaeppi et al., 2014), should be considered when comparing reports.

Few reports use *gyrB*, targeting the beta subunit of the bacterial gyrase (Barret et al., 2015; Bartoli et al., 2018), which allows the identification of the sequences to the species level and does not have a variation of copy number per genome. The comparison between *gyrB* and 16S rRNA markers showed essential differences in relative abundances (Barret et al., 2015). The use of PhyloChip, based on a 16S rRNA‐microarray, showed that Pseudomonadota was the predominant phylum in *A. thaliana* rhizosphere (Berendsen et al., 2018), surpassing by two the relative abundance of Bacillota and Bacteroidota, and by ten those of Cyanobacteriota, Actinomycetota, and Verrucomicrobiota, a pattern quite different of that reported using 16S rRNA next‐generation sequencing approaches (summarized in Poupin et al., 2023).

In addition, Regalado et al. (2020) compared rRNA amplicon analyses with metagenome shotgun sequencing and found unlike patterns. The discrepancies are produced because microbial abundances can be related to plant DNA abundances in the shotgun procedure, whereas amplicon analyses are based on microbial relative abundances. Therefore, microbial absolute abundances should be estimated relative to some specific plant component determinations (e.g., Durán et al., 2021; Wolinska et al., 2021). Recently reported is an improved approach based on host‐associated microbe PCR (hamPCR) to quantify microbial loads and describe community compositions (Lundberg et al., 2021). The hidden effects of experimental and data analysis variability lead to wrong conclusions, and the plant–microbe research community should address the need to systematize protocols and bioinformatic approaches (Figure 1B). Fortunately, recommendations for correcting biases and misinterpretations in sequence data analysis are available. For example, taxonomic profiling programs performed well at high taxonomic ranks, but parameter settings must be adjusted for lower‐rank analyses (Sczyrba et al., 2017). Another example is the importance of primer and sequence database selection in amplicon sequencing. However, the most critical aspects of shotgun metagenomic sequence analyses are assembly and binning, as these steps may introduce errors leading to misinformation (Lucaciu et al., 2019).

On the other hand, germ‐free plant models do not accurately represent typical plant characteristics (Partida‐Martínez & Heil, 2011). An essential part of publications on Arabidopsis microbiota indicates germ‐free conditions that are not consistently demonstrated. Sterile seeds, or those having very low, undetectable microbial counts, could be obtained when plant models have been grown for several generations under laboratory conditions. Thus, it is conceivable, but not systematically addressed yet, that the seed batches would have different microbial communities depending on the lab's time and conditions of maintenance. Truyens et al. (2016) demonstrated significant losses in the richness and abundance of the bacterial community after several generations of *A. thaliana* seeds grown in bacteria‐poor substrates, and similar results were obtained in *Setaria viridis* (Escobar‐Rodríguez et al., 2020). Recently, Sun et al. (2023) analysed the literature on the seed microbiota of wheat, rice, and maize, finding evidence for the role of these microorganisms in shaping plant performances. Therefore, part of the knowledge of the microbiota roles in plants is not correctly addressed if we do not consider the possible roles of microbial vertical transmission during sexual reproduction (Figure 1B).

Additionally, microbiota studies in plants other than *A. thaliana* still need to catch up in coverage and depth compared to this well‐known plant model (Marks et al., 2023; Poupin et al., 2023) (Figure 1B). As expected, studies on the microbiota of crop plants are mainly focused on relevant agricultural features and environmental stresses rather than on more fundamental grounds. See, as examples, recent reports or reviews on cereals (maize, rice, barley, Michl et al., 2023), wheat (Chen et al., 2022a; Michl et al., 2023), tomato (Oyserman et al., 2022), potato (Faist et al., 2023), and medicinal plants (Köberl et al., 2013). Expanding research across diverse plant species will help to uncover novel microbial interactions and mechanisms contributing to plant health, resilience to environmental stresses, and crop productivity.

In synthesis, methodological biases can be significantly controlled by introducing further systematization of protocols and including the seed compartment/vertical transmission component. In addition, including new plant models with features that are not present in Arabidopsis would significantly ameliorate current biases (Figure 1B).

### What microorganisms other than bacteria and fungi are we still looking for?

Plant microbiota studies are mainly focused on bacterial communities as they are far more abundant (10–1000 times) when compared to archaeal, fungal, and micro‐eukaryotic communities (Durán et al., 2018; Stringlis et al., 2018) (Figure 1C). Fungal communities have also been targeted mainly because of their role in plant diseases, both as pathogens and pest controllers (Priyashantha et al., 2023). The presence and potential function of archaeal members in plant microbial communities have been scarcely addressed (Borrel et al., 2020; Jung et al., 2020). This becomes more relevant, considering that the PCR primer sets used in most studies are not primarily defined to detect archaeal species. Despite that, Bressan et al. (2009) provided early evidence for the presence of archaeal species in Arabidopsis, and a low proportion of this domain was also observed in later studies (Bulgarelli et al., 2012; Lundberg et al., 2012). Furthermore, members of the Thaumarchaeota phylum have been described as relatively abundant in plant roots, surpassing bacterial phyla such as Nitrospirota or Bacillota (Tkacz et al., 2020).

Some studies have provided helpful information concerning eukaryotic microorganisms other than fungi (Hassani et al., 2018; Nguyen et al., 2023). For example, using PCR primers targeting Oomycetes and Cercozoa, Sapp et al. (2018) found that Oomycetes (Peronosporales) were not significantly different in soils compared to roots but discovered many groups of Cercozoa, and some of them were preferentially found in roots. Durán et al. (2018) described that *Pythium*, an oomycete genus, was significantly abundant in roots. Tkacz et al. (2020) found that the scarcely described microeukaryotes group is part of the Arabidopsis holobiont root microbiota. To aid in further protist studies, a collection of nearly 80 Cercozoa isolates was obtained from *A. thaliana* and is available for future research, including the design of SynComs (Dumack et al., 2021).

There is a paucity of reports on plant virome (see, for example, Aghdam et al., 2023) even though it is suggested that viruses not only cause diseases but also have commensal and mutualistic interactions, helping plants to overcome abiotic stresses (Poudel et al., 2021), and shaping plant ecology and evolution (Roossinck, 2015). Bacteriophages and temperate phages can additionally modify the ecology and evolution of plant‐associated microbial communities and should also be considered (Koskella & Taylor, 2018; Pratama et al., 2020; Tzipilevich & Benfey, 2021). The exciting possibility that a virome community forms part of the triad reported in the animal (human) holobiome, controlling the bacterial abundances, among other functions, has yet to be addressed in plants (Barr, 2019; Sessitsch et al., 2023).

Thus, plant microbiota research has overlooked microorganisms like archaea, viruses, micro‐eukaryotes, and those vertically transmitted during sexual reproduction (see above). To better comprehend the role of the different partners in these biological interactions, a broad focus might be needed in future studies (Figure 1C). In synthesis, it is essential to incorporate specialized research focused on archaea, microeukaryotes, and viruses to broaden our understanding of microbial players beyond bacteria. This approach will help elucidate their roles in plant–microbe interactions. (Figure 1C).

### How do microbe–microbe interactions and the plant environment shape microbial dynamics?

Little is known about how microbe–microbe interactions can influence the effects of a microbial community in a plant (Figure 1D). Conversely, how can the plant context change the microbial dynamic of the community compared to a plant‐free environment? (Figure 1, center). The research using correlational approaches (mainly looking for associations rather than causality) has progressed significantly over the last few years, primarily because of the use of molecular techniques based on high‐throughput (culture‐independent) sequencing, allowing the identification of the microbial metagenomes, taxonomic structure, diversity, and abundance in different plant niches. This strategy has been instrumental in revealing the influence of various factors on shaping plant microbiomes, including soil types (Bai et al., 2022; Bulgarelli et al., 2012; Lundberg et al., 2012; Schlaeppi et al., 2014), evolutionary changes (Schlaeppi et al., 2014), and host genetics (Bulgarelli et al., 2015; Johnston‐Monje & Raizada, 2011).

The use of SynComs has also helped to mimic, to some extent, the microbial ecological interactions occurring in the plant microbiome and for testing causal hypotheses in a more realistic context (Burghardt et al., 2018; Durán et al., 2018; Lee et al., 2020; Marín et al., 2021). Burghardt et al. (2018) studied strain competition's effect on legumes' nodulation. They inoculated legumes with a SynCom community of 101 rhizobia strains. Alternatively, they inoculated plants with each of the 101 strains. They found that nodulation was only weakly correlated with strain fitness in the single‐inoculation experiment. This suggests that competition among strains for host access and host preference influences selection on nodulation.

Additionally, Durán et al. (2018) analysed several SynCom inter‐kingdom combinations (fungal, bacteria, and plants), finding that Arabidopsis hosts a diverse community of filamentous fungi, with Ascomycota being the dominant group. However, when bacterial competitors were absent, consortia of root‐derived filamentous eukaryotes negatively affected plant health and survival. Shalev et al. (2022) studied the interactions among pathogenic and commensal *Pseudomonas* and how these interactions affected host health. They found that some strains rapidly induced adverse effects, killing plants when grown in axenic conditions. Interestingly, these adverse effects were diminished and slowed down in plants growing in non‐sterile substrates, emphasizing the importance of microbe–microbe interactions in the host responses (Figure 1D).

A few studies have attempted to define a core microbiota (the set of microbial taxa found in most plant species samples) (Bulgarelli et al., 2013; Vorholt, 2012). Defining these core microbiota enables the identification of a set of stable taxa with a greater probability of influencing plant phenotypes (Busby et al., 2017). Within these core microbiomes, the field still needs to address the ecological rules and the existence of keystone species (Paine, 1969) or functional redundancy (Walker, 1992). The keystone species hypothesis (Paine, 1969) assumes that if one species sustains a vital ecosystem function, it disproportionately affects the systems relative to its abundance. Conversely, the hypothesis of functional redundancy supposes that diverse organisms with similar or equivalent effects contribute to function persistence (Walker, 1992). The loss of a keystone species leads to a loss of function.

In the case of functional redundancy, there is potential for functional compensation after species loss by equivalent community members. Additionally, communities can have functional complementarity when members with different functions maintain community stability (Cardinale et al., 2011). Thus, changes in the community's taxonomic composition are not synonymous with function loss, which depends on the functional diversity or complementarity of the local assemblage. These hypotheses could also be applied to microbiota communities (Moya & Ferrer, 2016) and could account for a possible elasticity in the core microbiota community composition (Vandenkoornhuyse et al., 2015). In this context, Bai et al. (2015) studied the genome drafts of 400 isolates of different Arabidopsis microbiome niches, revealing that clusters of genomes are characterized by a relatively large core genome, with an average of 33.6% of the annotated proteins present in each member and a smaller fraction of singleton genes identified in only one genome per cluster (14.0%) (Bai et al., 2015). This evidence may support the functional redundancy hypothesis, but further functional analyses and comparisons with communities from other niches are needed.

Another aspect that may influence the role of different taxa in a community is its degree of stability or maturity. Using a leaf SynCom with 62 native bacterial members, Carlström et al. (2019) found that individual strains of specific taxa acted as keystone species. Furthermore, the timing and order in which keystone species arrive at the phyllosphere community determined the effects of groups or strains. In the early stages of the leaves' community establishment, removing specific taxa drastically affected the community structure. When introduced as later arrivals in a mature community, these dropped‐out strains or groups could colonize the plant phyllosphere but did not significantly affect its structure (Carlström et al., 2019). Also, they found that the order in which strains are introduced to the plant affects the outcome and how single strains drive the phyllosphere and rhizosphere community assembly (Bai et al., 2022; Carlström et al., 2019). Similar results were obtained in an Arabidopsis field assay, where leaf microbial communities varied at the beginning of the plant growing season, becoming less variable as the season progressed, showing conserved temporal patterns (Almario et al., 2022). These results suggest that an initial community is challenging to perturb once established, and intentional microbiota manipulations should be more effective if applied when the microbial community of the host is still developing.

Competition for niche has also been described as one of the factors that can shape a plant microbiota. Schäfer et al. (2023) combined experimental data with genomic models to predict bacterial interactions in the phyllosphere. They found that carbon utilization, niche partitioning, and cross‐feed mechanisms are critical in determining the microbial interactions in this oligotrophic niche. Nevertheless, the environments where microbe–microbe interactions occur (i.e., phyllosphere and rhizosphere) are highly heterogeneous in space and have physicochemical properties (Figure 1, centre). However, most analyses consider them as a single research unit. Schlechter et al. (2023) recently studied the role of resource competition using a model of an epiphyte from the phyllosphere (*Pantoea eucalypti* 299R) and six strains from two different phyla. They found that the metabolic resource overlap is more closely related to competition in homogeneous environments than in the phyllosphere. They also observed that macro changes at the whole‐leaf scale do not accurately represent competition phenomena at the local level (Schlechter et al., 2023). They propose that factors other than metabolic niches, such as motility and production of inhibitory compounds, could explain microbial competition. Getzke et al. (2023) analysed the impact of the exometabolite output on binary competition between different strains from the *A. thaliana* rhizosphere. They observed a higher inhibitory activity in root‐associated isolates than in soil‐associated ones.

These findings highlight the significant role of bacterial competition within the rhizosphere, which offers a richer resource environment than the phyllosphere or bulk soil. They also suggested that the capacity to produce pyoverdine (an iron chelator) could be crucial in determining the ability of *Pseudomonas* to outcompete other strains for iron scavenging in the rhizosphere. Interestingly, Jiang et al. (2023) found that a SynCom composed of native bacteria outperformed commercial rhizobacteria in promoting maize growth under low‐fertility conditions. This was attributed to effective colonization and positive interactions with the resident microbial community. Regarding microbial competition within the plant microbiota, most studies have obtained interesting data from studying binary interaction schemes (between two strains or microbial species).

Plantontology's importance in influencing plant microbiome development has also been discussed (Figure 1, centre), finding that the microbial communities of different plant niches change during plant growth (Chaparro et al., 2013; Edwards et al., 2018). Edwards et al. (2018) studied the life cycle in field‐grown rice over multiple seasons and locations, finding that root microbiota composition varied not only with chronological age but also with the developmental stage of the plants, with a significant compositional shift correlated with the transition from juvenile to adult plant phases. Moreover, Chen et al. (2019) found that the root‐released organic carbon varied across different plant stages, with a more substantial influence on bacteria than on the composition of the fungal community. Furthermore, Beilsmith et al. (2021) found a more decisive influence of plant tissue type and developmental stage on assemblage composition than that of geographic site. Likewise, the phyllosphere tissues housed increasingly distinct microbial assemblages as plants aged, stressing the role of host development in plant microbiome shaping.

Moreover, the leaf side also influences the phyllosphere microbiome, hosting different plant–microbe interactions (Smets et al., 2023). Therefore, it is essential to consider that microbial ecological interactions influence the phenotypes observed in plants. The plant's development and characteristics also impact the population dynamics of its microbiota (Figure 1D and centre). In synthesis, future research on plant microbiome dynamics should incorporate ecological aspects such as competition/collaboration among microbial players and the role of the plant context (developmental stage, stress response, and ecotype) (Figure 1D).

### How do we assess plant–microbiome systems as complex ones?

Plant and their microbiota are complex systems as the outputs are not the sum of the parts, and multifactorial approaches should be addressed (Figure 1E). Land plants have interacted with microorganisms since colonizing terrestrial habitats (Field et al., 2015; Fitzpatrick et al., 2018). However, how these interactions have shaped host plant evolution, ecology, and the assembly of plant microbiomes in macroevolutionary timescales has yet to be discovered. Also, how the environment can modulate these interactions has yet to be understood (Figure 1). For instance, it has been reported that abiotic stresses such as drought or nutrient limitations can induce shifts in plant microbial communities (Hacquard et al., 2015; Naylor et al., 2017). In this context, the setup of experimental conditions to induce stress in plants often varies from experiment to experiment (e.g., salt concentration and level of drought), making it difficult to compare all reports (Yang et al., 2023; Zhang et al., 2024).

Fitzpatrick et al. (2018) compared the microbiota (rhizosphere and endosphere) of 30 phylogenetic diverse angiosperms. They found that the rhizosphere showed higher diversity and greater evenness in abundance than the endosphere. Also, a phylogenetic link was observed with the endosphere microbiota but not those from the rhizosphere (Fitzpatrick et al., 2018). Interestingly, they also found that patterns of root microbial recruitment among host plants in both the endosphere and rhizosphere were associated with competitive interaction among plants, emphasizing microbiota's role in plant ecology (Fitzpatrick et al., 2018). The role of intraspecific host variation in shaping the plant microbiome has recently started to be addressed (Shalev et al., 2022; Thiergart et al., 2020). Thiergart et al. (2020) monitored root‐associated microbial communities in *A. thaliana* and co‐occurring grasses in 17 European sites across three years. They found that the primary driver for microbial communities at these large scales was the soil type and that the host genotype effects were minor. Nevertheless, Shalev et al. (2022) analysed geographical changes on a smaller scale, using six *A. thaliana* genotypes sampled from a maximum of 40 km apart in the same geographic region and from the same host genetic group. Contrary to what was expected, they found that host genotypes had a minor but significant effect (5%–12%) on the compositional variation of different tested SynComs.

Microbial capabilities are commonly assessed based on their metabolic behaviours under specific scenarios or due to genetic determinants. However, it is crucial to recognize that microbial responses can also exhibit phenotypic plasticity in response to varying environments (Figure 1, centre). Hemmerle et al. (2022) explored this phenomenon by studying two leaf‐associated bacteria belonging to the Sphingomonadaceae and Rhizobiaceae, with predicted niche overlap based on mono‐inoculation experiments. Interestingly, when co‐inoculated in leaves, these strains changed their protein production patterns, which increased their dissimilarities and allowed their co‐existence in plants.

Many plant responses to the environment are mediated by circadian regulation (Grundy et al., 2015; Panter et al., 2019). Circadian regulation of the plant microbiota has also been reported (Hubbard et al., 2018). These authors found that the rhizosphere community structure of Arabidopsis varied between day and nighttime points, and clock misfunction significantly affected rhizosphere communities.

Epigenetic changes are pivotal in plants, controlling gene expression and helping plants adapt to biological and environmental stresses throughout their growth and development (Chinnusamy & Zhu, 2009; Stroud et al., 2013; Thiebaut et al., 2019). Unfortunately, knowledge of the epigenetic effects of microbiota or single beneficial strains in plants is scarce, and only a few articles report them (Vilchez et al., 2020; Wilkinson & Ton, 2020). For instance, Chen et al. (2022b) inoculated a perennial herb (*Phytolacca americana*) with two beneficial strains of *Bacillus*. They found that inoculation has minor effects on the rhizosphere microbiota but significant effects on plant gene methylation, which was associated with the promoting effects of the strains.

In future research, a comprehensive understanding of plant–microbiome interactions should encompass various dimensions of the plant's dynamics, including chronological time, phenotypic plasticity, the circadian cycle, plant‐life history traits, epigenome, and evolutionary and ecological considerations. Moreover, concurrent multiple layers of the abiotic and biotic aspects of the environment should also be considered. This holistic approach is essential for unravelling the full complexity of these interactions (Figure 1E and centre).

## CONCLUDING REMARKS

We propose several key considerations to advance our understanding of plant–microbiome interactions. Firstly, microbial taxa alone may not be accurate predictors of microbiota functions; thus, there is a need for functional assessments of microbiomes. Moreover, methodological biases in plant microbiota research should be systematically addressed to ensure robust comparisons. Additionally, overlooked microorganisms like archaea, fungi, and viruses warrant greater attention. Also, understanding how microbial dynamics (competition/cooperation) in local micro‐niches and natural communities affect plant performances remains a crucial area of study. Finally, we propose that the plant holobiont should be studied as a complex system that emerges from intricate interactions of multiple layers, where the constituent parts determine the identity of the whole. Still, the whole also affects the identity of the parts (Figure 1). Interdisciplinary efforts should be fostered to explore these complexities deeper, leading to reliable and effective microbiome‐based solutions. The most critical issue to be addressed is effectively communicating the value of improved comprehension of plant–microbe holobiont and their connections to climate change adaptation and sustainable production. Not immediately, but shortly, initiatives like International Microbiology Literacy (IMiLi, https://imili.org) would pave the way.

## AUTHOR CONTRIBUTIONS

**María Josefina Poupin:** Conceptualization (equal); formal analysis (equal); funding acquisition (equal); investigation (equal); methodology (equal); project administration (equal); validation (equal); writing – original draft (equal); writing – review and editing (equal). **Bernardo González:** Conceptualization (equal); formal analysis (equal); funding acquisition (equal); investigation (equal); methodology (equal); project administration (equal); resources (equal); validation (equal); writing – original draft (equal); writing – review and editing (equal).

## CONFLICT OF INTEREST STATEMENT

The authors declare no conflict of interest.
